# Review on V2X, I2X, and P2X Communications and Their Applications: A Comprehensive Analysis over Time

**DOI:** 10.3390/s19122756

**Published:** 2019-06-19

**Authors:** José Manuel Lozano Domínguez, Tomás Jesús Mateo Sanguino

**Affiliations:** Department of Electronic Engineering, Computer Systems and Automatics, University of Huelva, Av. de las Artes s/n, 21007 Huelva, Spain; jose.lozano@diesia.uhu.es

**Keywords:** 5G, autonomous and connected vehicle, communication technology, cybersecurity, infrastructure-to-everything, literature review, pedestrian-to-everything, safety, smart city, vehicle-to-everything

## Abstract

Smart cities are ecosystems where novel ideas and emerging technologies meet to improve economy, environment, governance, living, and mobility. One of the pillars of smart cities is transport, with the improvement of mobility and the reduction of traffic accidents being some of the current key challenges. With this purpose, this manuscript reviews the state-of-the-art of communications and applications in which different actors of the road are involved. Thus, the objectives of this survey are intended to determine who, when, and about what is being researched around smart cities. Particularly, the goal is to situate the focus of scientific and industrial progress on V2X, I2X, and P2X communication to establish a taxonomy that reduces ambiguous acronyms around the communication between vehicles, infrastructure, and pedestrians, as well as to determine what the trends and future technologies are that will lead to more powerful applications. To this end, this literature review article presents a comprehensive study including a representative collection of the 100 most cited papers and patents published in the literature together with a statistical bibliometric analysis of 14,364 keywords over 3422 contributions between 1997 and 2018. As a result, this work provides a technological profile considering different dimensions along the paper, such as the type of communication, use case, country, organization, terminology, and year.

## 1. Introduction

**Figure 1:**
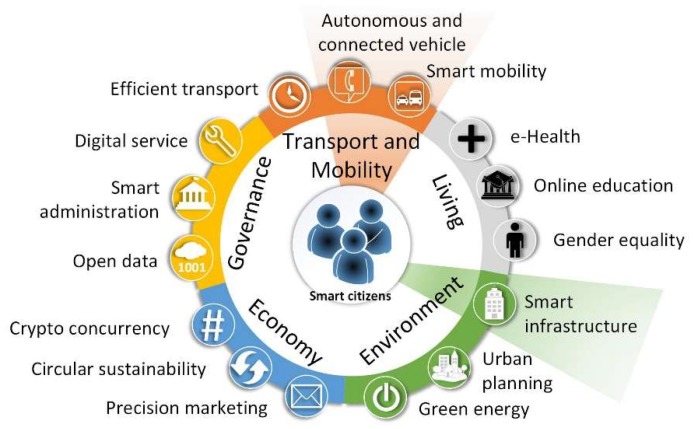
Person-centric adaptation of the Smart City Wheel proposed in [1]. The topics shaded in orange and green concern this research.

Smart cities are scenarios of innovation, challenge, and opportunity where the information and communication technologies (ICTs) are being exploited at the service of people to improve economy, environment, governance, living, and mobility (Figure 1). Investment in smart cities is growing, evolving from small projects to great technological market opportunities around universities, governments, and industries [1]. As a consequence, global spending on emerging technologies for the progress of the smart cities reached $80 billion in 2018 and will progressively increase up to $135 billion by 2021 according to a report made by IDC Research [2].

Most of the investment is devoted to intelligent connected transport (ICT) and sustainable mobility, followed by smart lighting and environmental monitoring with cross-country variations. Specifically, the US, Japan, and Europe invest in transport and mobility first (e.g., driving safety, traffic efficiency, or telematics services), China spends more in video surveillance systems (e.g., facial recognition or license plate recognition), while environmental monitoring (e.g., water, waste, or air pollution) will be relatively more important in Japan [3]. From this, the US and China are currently the two largest markets for smart city technologies with $22 billion (annual growth rate of 19%) and $21 billion (annual growth rate of 19.3%), respectively [2].

Smart cities are often referred to as digital or connected cities since they implicate the intelligent use of technology to add value and attain more efficient services (e.g., to alleviate the problems resulting from the massive urbanization and population growth). A key aspect of a smart city is the use of sensing, communication, and social capabilities as part of a wider concept around the Internet of Things (IoT) [4]. This approach has been made possible by providing intelligence, mobile sensing, and wireless capability to infrastructures (e.g., green buildings), persons (e.g., wearable devices), and vehicles (e.g., intelligent transport systems) to facilitate data access, which is fundamental to make smart cities a reality [5,6,7].

According to a recent study, the IoT market (i.e., manufacturing, transport, logistics, and public services) will increase the investment to spend $123.8 billion on IoT platforms and services by 2021 [8]. For instance, the Spanish market achieved 5 million connected objects in 2018 and this is expected to increase up to 8 million IoT lines in 2022 (annual growth rate of 10.9%) [9]. That growth, in line with other EU countries such as France, Germany, Sweden, or the UK (up to 1.3 billion connected objects worldwide) is mainly being demanded by the personal market and the industry 4.0 (i.e., financial, banking, retail, security, transport, logistics, and automotive sectors). 

In particular, IoT is pivotal in transforming classic transport and automotive services into intelligent transport systems (ITS) by enabling detection (e.g., image or video), artificial intelligence (e.g., sensor fusion), and data processing (e.g., big data and analytics) for autonomous and/or connected vehicles [10]. In this area, vehicular communications are gaining primary attention from both research community and industry, where vehicles are the third type of connected device with the greatest growth potential, after smartphones and tablets [11]. In this sense, the total average cost of implementing connected vehicle technology in the US is projected to increase from ~$1.2 billion to ~$3.75 billion in 2022 with investments of ~$2.9 billion annually from 2025 [10].

### Outline

The current scenario of investment, research, and development described above is being traduced in numerous manuscripts and patents published in leading scientific journals, conferences, and intellectual property databases. When referring to vehicular communication, the research includes a very rich literature, but mostly oriented to cover specific aspects of the technology. As an example, this is the case of ad-hoc networks [12], information management systems [13], security [14], and access technologies [15] around vehicle-to-everything (V2X) communication published in 41 review and survey manuscripts between 1997 and 2018 (i.e., secondary research). 

Nonetheless, there is hardly a mention in the bibliography—neither primary research nor secondary research—to other types of communication, such as infrastructure-to-everything (I2X), pedestrian-to-everything (P2X), and their variants. The main reason is that they stand for more emerging markets and do not enjoy yet a standardized definition in the state-of-the-art to be used by the research community (i.e., there is no uniformed consensus about these terms). Moreover, as far as we know, most surveys do not always emphasize on the historical evolution (i.e., they are frequently limited to specific aspects and/or contemporary technologies), do not cover statistical profiles, and do not conduct bibliometric studies with a broad perspective of the context over time. 

For this reason, the research question this paper aimed to examine was: What are the past, current, and future interests in V2X, I2X, and P2X communication? It had four main objectives:

1. To situate the industrial and scientific progress through the examination of who, when, and about what the research has been done on emerging technologies for smart cities, especially focused on V2X, I2X, and P2X communications;
2. To systematically collect features on the type of vehicular communication, field of study, technologies, and applications to establish a time reference frame on significant characteristics;
3. To undertake a comprehensive bibliographical analysis on the relationship of the publications comparing the most productive countries and organizations along time, as well as to the inference of an emerging technology over other;
4. To review what future milestones lead technologies to more powerful applications on V2X, I2X, and P2X.

To this end, the present paper is designed as a review article—which is not a primary research article—and structured as follows. The following section provides a taxonomy on V2X, I2X, and P2X communication and their variants. Then, the paper describes the recent attention attracted from governments, academics, and industries around V2X, I2X, and P2X applications. As a result, a summary table about the profile of the most representative applications present in the R&D literature is provided in Appendix A. The next section provides a contextual bibliographic study on the most influential technologies around smart cities. Finally, the paper presents the future trends and emerging technologies to reach the conclusions.

## 2. Taxonomic and Technical Analysis

Although the term for communication between machines (M2M) or devices (D2D) is a traditional and clear concept for the entire research community, it does not present always clear definitions when applied to vehicles, infrastructure, or pedestrians. As an example, the terms for car to car (C2C), vehicle to vehicle (V2V), car to everything, or car to all (C2X) are used indistinctly as vehicle to everything (V2X) communication in [16,17,18], among others. This is even more ambiguous when the different actors are included in the interaction but the origin of the data source is not properly considered in the communication process. Therefore, it is necessary to deepen their definitions and make a classification of the various existing forms.

The taxonomy proposed below aims to correct errors, to provide further clarification on frequently misunderstood concepts and to include new acceptations such as for the infrastructure-to-everything (I2X) and pedestrian-to-everything (P2X) communications. To this end, the taxonomy has been elaborated based on the full spectrum of terms collected around V2X, I2X, and P2X communications. Therefore, we provide functional definitions for the different variants. This classification does not intend to provide specifications or impose technical requirements on the various types of communication, but to harmonize terms with the following purposes:

1. To provide clarity and stability regarding the role played by the V2X, I2X, and P2X communications;
2. To provide a useful framework that saves time and effort during the development of specifications and technical requirements (e.g., in standards);
3. To respond to issues of scope for the writing of future regulations, laws, and policies.

**Figure 2:**
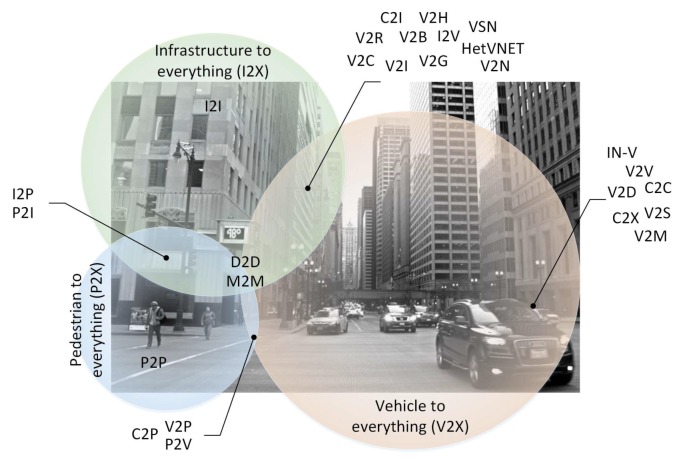
Diagram of interactions between vehicle, infrastructure, and pedestrian communications.

As can be appreciated in the following section about the state-of-the-art, the different types of communications can be classified as V2X, I2X, and P2X depending on the origin of the communication. Then, the following taxonomy is proposed (Figure 2).

- **Vehicle-to-Everything** (V2X): Communication from vehicle to any entity, which includes in-vehicle connectivity (IN-V) with sensors (V2S) or other onboard devices (V2D) such as infotainment systems. This definition often encompasses the terms for car to all/car to everything (C2X), car to car (C2C) or vehicle to other vehicles (V2V) such as motorcycles (V2M). This classification group also includes other more specific types of interactions, such as vehicle to grid (V2G) to communicate with smart grids to receive or return electricity, car to infrastructure (C2I) or vehicle to infrastructure (V2I) to communicate with the road (V2R), such as road side units (RSUs) acting as stand-alone units or relay nodes that provide safety and traffic updates (e.g., traffic lights), vehicle to networks (V2N), heterogeneous vehicular networks (HetVNET) or vehicular sensor networks (VSN). These last also include vehicle to broadband cloud (V2B) or vehicle to cloud (V2C) communications utilized for software upgrades or information updates. V2I also includes vehicle to home (V2H) appliances such as lighting or air conditioners, while the car to pedestrian (C2P), vehicle to pedestrian (V2P), or vehicle to phone (V2P) communications may include smartphones and wearables worn by persons. Note that the term vehicle-to-home must be disambiguated with respect to the term vehicle to humans (V2H).
- **Infrastructure-to-Everything** (I2X): Communication from infrastructure to any entity, which may include other infrastructures (I2I), vehicles (I2V), or pedestrians (I2P). This term must be disambiguated with respect to the term individual to individual (I2I).
- **Pedestrian-to-Everything** (P2X): Communication from pedestrian to any entity including other pedestrians (P2P), infrastructure (P2I), and vehicles (P2V). Note that these terms must be disambiguated with respect to the peer to peer (P2P) and payment to individual (P2I) approaches.

### Technological Profile of the Survey

As stated in the previous section, there is no developed methodology to uniformly divulgate the development of applications based on V2X, I2X, or P2X communications. Nevertheless, it is possible to elaborate a set of basic features that they must fulfill. In order to assess one of the contributions made by this work to the field of V2X, I2X, and P2X communications, a comparison of the characteristics and capabilities including a set of 100 representative manuscripts collected in this review is shown in Table A1 (Appendix A). The followed methodology uses the number of citations as criteria to select the most representative papers based on the guidelines to make a literature review mentioned in [19]. Although the criteria could cause a potential bias in the results, since the number of citations increases with time and, thus, the selection could disqualify the most recent papers in the area, we have obtained the following distribution: 20.79% of the documents were published between 2000 and 2009, 45.54% of the documents were published in the period 2010–2013, and 33.67% were published between 2014 and 2018. This means that one third of the papers were published in the last five years, giving a significant insight on the current state-of-the-art.

**Figure 3:**
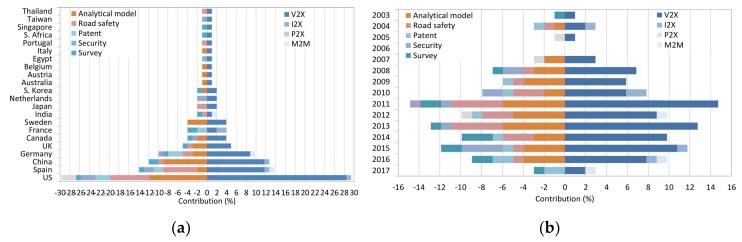
For a representative sample of the 100 most cited publications in the state-of-the-art: (**a**) Type of communication and use case versus country, and (**b**) type of communication and use case versus year.

The analysis about the current research shows that the most productive country is the US (29.7%), with the study of analytical models for vehicular communications (11.88%) and road safety applications (7.92%) being its main contributions (Figure 3a). The next largest contributor is Spain (13.86%), which is mainly focused on road safety applications (6.93%), followed by China (11.88%) in the study of analytical models (8.91%). On the other hand, 2011, 2013, and 2015 were the most representative years (14.85%, 12.87%, and 11.88%, respectively) with contributions in analytical models (15.84%), road safety applications (9.9%), and cybersecurity (3.96%). Moreover, the results show that V2X is the type of communication most investigated (92.14%), with 2011, 2013, and 2015 being the most productive years (14.71%, 12.75%, and 10.78%, respectively) (Figure 3b). I2X is the second type of communication most studied (4.9%), focusing its contributions in 2004, 2010, 2015, and 2016 (0.98%, 1.96%, 0.98%, and 0.98%, respectively). Finally, P2X has barely been studied, only becoming a field of study in recent years (1.96%) where 2016 and 2017 were the main years of contribution (0.98% and 0.98%, respectively).

## 3. State-Of-The-Art

The purpose of this section is to present a historical review of the most representative technological initiatives that have promoted the development of different V2X, I2X, and P2X approaches over time, from the early regulations carried out by governments and regulatory bodies to the current research and development (R&D) being performing by industries and universities.

### 3.1. Government and Regulatory Agencies

The history of connected cars dates officially from 1996 with OnStar, a company created by General Motors—collaborator of Electronic Data Systems and Hughes Electronics Corp—whose goal was to request medical help by routing a phone call to an emergency center after a car accident [20]. The e-Call system, which initially started as a voice service activated by airbag deployments only aboard some Cadillac models, has evolved nowadays into a complete sensor-based automatic crash response system capable of determining the severity of the car impacts. To this end, the system comprises GPS location, remote diagnosis, network access device, 4G, and WiFi spot to process up to 5 million phone calls per month in the US.

This milestone in the field of the ITS development has been able to progress thanks to several facilitating entities such as the US Department of Transportation (DOT) and the Federal Communications Commission (FCC) who regulated in 1999 the use of a 75 MHz band in the 5.9 GHz spectrum (i.e., 5850–5925 MHz) for unlicensed access technologies [17]. This effort was preceded by Japan, whose country reserved its radio spectrum for ITS applications in the 760 MHz and 5.8 GHz frequency bands after initiating its deliberations on regulation policies by 1994 [21]. Similarly, the EU and the European Telecommunications Standards Institute (ETSI) allocated in 2008 a 30 MHz band for safety-related applications of ITS in the same spectrum region (i.e., Commission Decision 2008/671/EC). This activity was also followed by other countries in the Asia Pacific region, such as Korea, Singapore, China, and Australia, who defined their spectrum allocations between 2016 and 2017 [22].

Meanwhile, the Institute of Electrical and Electronics Engineers (IEEE) formed in 2004 a task force to work in an IEEE 802.11-based draft for wireless access in vehicular environments (WAVE) that resulted in the IEEE 802.11p amendment by 2010. It was the basis for the future European standard for cooperative ITS environments—such as the one used in the V2V and V2I communications—where the first version was released in 2014 by ETSI and the European Committee for Standardization (CEN) as ETSI ITS-G5. IEEE 802.11p was integrated with IEEE 1609 and SAE J2735 to provide a complete standardized message protocol stack, which was considered by DOT in 2012 for dedicated short-range communications (DSRC) in vehicle-based applications (e.g., toll collection, emergency vehicles, road works, braking warnings, etc.).

Consequently, the EU announced in 2010 that ITS applications were already interoperable (2010/40/EU) and started working by 2014 in a regulatory framework to improve some key areas in V2X technology such as cybersecurity and radio interferences. At the same time, the US National Highway Traffic Safety Administration (NHTSA) published a report in 2014 stating that the V2X technology was technically proven and ready for real deployment in markets. Additionally, China formally proposed in 2015 its own national development program for intelligent connected vehicles (ICV), which has to be finished in 10 years according to its strategic plan known as Made in China 2025 [15]. As a result, this progress has led some countries to mandate all vehicles to carry V2V technology in order to reduce collision-based accidents (e.g., EU from 2018 and US from 2021) [10].

While DSRC proved to be an adequate ad-hoc technology enabling V2V applications due to the short range (300–1000 m), low latency (200 µs), and medium data transmission rate (27 Mbps), cellular networks help to support V2I solutions due to the broad spread and commercial success of the mobile communications. Cellular V2X (C-V2X) has been standardized since 3G/UMTS and 4G/LTE proved to be useful (i.e., up to 2 km, 1.5–3.5 s, and 75–300 Mbps) but not versatile for time-critical scenarios. In this sense, the Third Generation Partnership Project (3GPP) has been a standardization body especially active in the development of 5G technology. It has regulated C-V2X from releases 14 and 15 in early 2016 and early 2019, respectively [23,24].

According to the previous scenario, although the major shift lever for the development of ITS applications has been the regulation of the radio spectrum and the technology standardization by the various regulatory bodies, there are still some limitations that hinder worldwide adoption. On the one hand, the locations of the radio bands are unique to each country and not interoperable across different territories (e.g., the Japanese ITS system in the 760 MHz band overlaps with the 4G/LTE mobile network operating in New Zealand) [25]. On the other hand, the degree of investment required by some other applications (e.g., V2I) will take time to be implemented due to the numerous infrastructures existing [26]. This means a different adoption rate of LTE versus DSRC-based solutions. For all the above, an increase in the efforts of governments, regulatory bodies, industries, and scientific community is still necessary due to the ambiguity of the communication technologies, lack of supporting infrastructures, overall implementation cost and attention to certain technical challenges, such as the improvements in cybersecurity [27].

### 3.2. Primary Research in Literature

The work of many governments around the world to legislate wireless technologies is still underway to support V2V and V2I communications, thus being responsible for facilitating and regulating the development of the automotive industry in the coming years [13,28]. This regulation must support the agreements reached in the standards of the different regulatory agencies of each geographical area. In line with this activity, there are studies in the state-of-the-art that provide a broad vision about the relationship of wireless communications in vehicles and other actors on roads, such as infrastructures and pedestrians [29]. The most cited papers found in the literature were initially concerned with the study of different communication approaches, technological challenges, and their applications. This is the case, for instance, of the IEEE 802.11 standard adopted in DSRC communication [30,31]. In this sense, the interest on the proposal of a bandwidth of 10 MHz in the 5 GHz band for vehicular communications based on the IEEE 802.11p standard stands out [32,33,34,35]. 

One of the most analyzed aspects in the history of vehicular communications is the propagation of wireless signals in the 5 GHz band, as well as how different factors affect V2V and V2I communications. Some of the most studied factors include the vehicle density, relative speed between vehicles, and average vehicle speed [36,37,38]. In addition, the distance between transmitter and receiver as well as the line-of-sight (LOS) occluded by both stationary and moving vehicles were other factors studied [39,40,41,42,43]. In order to understand and improve these concerns, both theoretical and experimental studies were carried out. One example of this is the characterization of the signal loss through stationary and non-stationary models made in [44], while a new method that offers better approximations to determine this signal loss was exposed in [45]. Some authors use geometric modeling since it provides more accurate results for mobile and stationary objects (e.g., vehicles, trees, and buildings) than traditional signal propagation models [46,47,48]. There are also other authors who take advantage of radio-cognitive techniques for the study of the spectrum as these techniques provide precise information. This is the use case of vehicles that circulate on public roads whose information on the spectrum is shared by other vehicles. The availability of the spectrum allows to know future positions of vehicles [49,50]. Also related to the communication aspects, other studies have determined that the Doppler effect should be taken into account in vehicular communications because different reflections of the same signal can be received in different times and interfere [51]. Therefore, a new approach to palliate Doppler interferences based on a new inter-carrier scheme more efficient and less complex than previous used schemes is presented in [52]. Other authors have proposed the use of RADAR integrated in vehicles—as transmitters and receivers in V2V communication—in conjunction with conventional communication units in order to improve communication performance through the transmission of modulated information in the 24 GHz or 76 GHz spectrum provided by RADARs [53]. Nevertheless, other proposals go beyond the transmission based on radio frequency (RF) waves and propose the use of car headlights and taillights as a means of communication. As the main drawback, the use of light in the visible spectrum limits the range of communication with other vehicles to the cover range of the car lights [54].

Another field of study extensively analyzed by researchers has been the IEEE 802.11p standard. The state-of-the-art includes many proposals of improvement with respect to this standard since it is the most used in vehicular communications. The improvements mainly focused on the media access control (MAC) layer due to the high mobility of the network actors [55], which can cause failures in the estimation of communication channels and a decrease in network reliability [56]. This part of the network is responsible for guaranteeing fast, reliable, and collision-free access to the medium in vehicular ad hoc network (VANET) applications, as mentioned in [57,58,59]. These documents also proposed that media-access protocols should follow a time division multiple access (TDMA) scheme rather than the scheme currently used based on carrier sense multiple access (CSMA), because TDMA offers better performance in delivering packets on time than CSMA. Improvements to the current standard include the utilization of contention windows [60], a better estimation of communication channels [61], as well as the improvement of the performance of the MAC layer to minimize the bit error rate [62]. To solve these problems, Eichler, Ma and Zhao [30,31,63] offer a new perspective to solve the problems detected in the MAC layer (e.g., MAC congestion control to avoid collision or channel estimation through channel interleaving and channel coding). Despite all these advances, not all problems have yet been solved or are not yet perceived by users as advantages [12].

Although IEEE 802.11p is the most widespread standard in vehicular communications, it is not the only field of R&D. Cellular networks are also relevant for the vehicular communication because they may offer better performance in some cases than the 802.11p-based networks [64,65]. Other viewpoints considered by some authors argue that the access technologies should be selected on the basis of the vehicle speed [66]. This approach uses LTE for V2I communication and IEEE 802.11p for V2V communication [67,68]. The combination of both approaches, known as HetVNET, is a better solution than separately. According to a study, the use of HetVNET networks requires further research and development of new network topologies, as well as better network selection schemes (i.e., effective vertical handover techniques) [69]. Therefore, there are other works that propose the use of device-to-device (D2D) communication to support vehicular communications, where schemes promise suitable performances for V2V communications as mentioned in [70].

In addition to the communication models based on IEEE 802.11p or cellular networks, there are authors who propose other network approaches. An example is Name Data Networking (NDN), where the main advantage is the fast exchange of information between vehicles (i.e., V2V) or vehicles and infrastructure (i.e., V2I). As the main drawback, NDN requires an adequate density of vehicles and low distance between the participants according to [71,72]. Apart from that, other authors claim a network model based on the Software-defined Networking (SDN) and fog computing paradigms due to the higher flexibility, scalability, location capability, and fewer delays than the current network models [73,74]. Another point of view preferred by other authors to develop their communication approaches is the use of position-based routing protocols as they offer more performance in highly dynamic mobile networks [75]. 

Once the main technologies used in this area have been described, a set of additional outstanding papers are grouped below by the use case to which they belong.

#### 3.2.1. Road Safety

Road safety is one of the main use cases of vehicular communications since it allows saving lives and avoiding injuries to the vehicle occupants. For this purpose, road safety admits research on problems related to the safe access to highways, secondary roads, or areas with reduced visibility, as well as research on traffic congestion management. Several noteworthy examples in the literature describe a protocol based on emergency warning messages (EWM). As the main advantage, this approach has a low delay constraint to ensure the reception of the messages on time, which has been utilized to avoid collision-based accidents on motorways [76]. Similarly, a protocol that prevents network congestion so that emergency notifications sent to users arrive on time was presented in [77]. To achieve this, the protocol exploits the chain effect and removes redundant messages. Moreover, various systems that allow the prediction of traffic congestion in an area and then warn users were developed. On the one hand, approaches based on cooperative vehicular communication techniques were presented in [78,79]. On the other hand, algorithm-based solutions to detect situations of congestion were developed in [80,81]. However, other authors have focused their works on avoiding collisions in urban intersections using V2V communication. Some of them solve this problem by means of Fuzzy logic as a control mechanism [82,83], while others prefer algorithms based on formal theoretic methods [84]. 

There are also different proposals with the aim of increasing passenger safety. One of them is an RFID-based application whose signal is recognized to adapt the vehicle speed to the road [85]. Another application is a distributed cruise control that adapts the car speed in function of the road status [86]. One more is a system that facilitates the incorporation of vehicles to a main road coming from a secondary one by adapting their speeds [87]. In this sense, a speed control system that improves the road flow by taking account of data from accelerations and decelerations of nearby vehicles was shown in [88]. 

Another perspective analyzed in research by some authors has been ecological driving, a strategy that adjusts the vehicle speed so that the total fuel consumption around an intersection is optimized through the use of V2I communication [89]. Another field also exploited through different approaches is that concerning traffic monitoring systems. The first one analyzes routes and helps in decision-making through cloud computing so that drivers avoid traffic congestion [90]. The second one utilizes IoT and machine-to-machine (M2M) communication to create maps of road conditions based on data shared from in-vehicle smartphones [91]. Another aspect examined in this field is the real-time driving assistance system. One solution is based on cameras and V2V communication, which prevents vehicles from becoming obstacles to the drivers’ field of view (FOV) in a vehicle platoon [92,93,94]. Other examples are based on various planning methods that allow autonomous vehicles (AVs) to analyze possible maneuvers, select the safest movements, and determine the best trajectories to achieve their destinations [95]. Finally, an adaptive traffic control system that improves road flow by preventing long queues of vehicles intended to cross a given intersection was presented in [96]. This system also includes strategies for special vehicles such as ambulances or fire trucks.

In summary, all the examples previously collected make mention to V2V or V2I communications. Nevertheless, none of them involve vehicular communication with pedestrians or similar. At the moment, V2P communication is an emerging technology with great potential being developed, not only to improve the safety of the road users, but also to improve the efficiency of the traffic flow. To cite a few, some potential applications based on V2P communication can be found in [97,98].

#### 3.2.2. Cybersecurity

Another important aspect extensively studied in vehicular communications is security, since all vehicles can be exposed to security breaches that can produce fatal consequences to the occupants [99,100]. Several approaches in V2X communication are listed in [101] and [16], where the most used is the public key scheme [102,103,104,105,106] as it provides integrity and authentication through the IEEE 1609.2 standard [16]. In this field, other techniques such as the group signature [107] or the symmetric authentication schemes [108] also stand out but are less used as they are not part of the IEEE 1609.2 standard. However, the LTE authentication and key agreement protocol (LTE-AKA) is proposed to protect communications against possible attacks when 4G/LTE—or the coming 5G—is used as an access technology [11]. Beyond these studies, there are other novel works aimed at developing secure approaches applied to specific scenarios such as Tesla++, a protocol suitable for minimum data transmission and low energy consumption that prevents and protects against denial of service (DoS) attacks in VANETs. Despite of the deep understanding on security, there are aspects still unresolved. Among the most important for VANETs is to evaluate the reliability of the nodes that communicate through the network, to detect and revoke the trust on a rogue node, to guarantee the security and/or privacy—in terms of the vehicle traceability—as well as to detect malicious software [14].

#### 3.2.3. Commercial Applications

Vehicular communications, in addition to increasing road safety in the various ways described above, also provide a new approach for multimedia broadcast services and applications (MBMS). It is noteworthy that the development of new technologies, such as the vehicular communications, will greatly help the multimedia content to migrate to mobile platforms [109]. An example of this new contribution is the ability of communications to support streaming video while vehicles circulate on public roads [110]. This is possible thanks to the combination of real-time and non-real-time data; the first one is used to send inter-vehicle messages for safety purposes while the latter is used to transmit multimedia content (e.g., video or audio) [111].

#### 3.2.4. Other Directions of Research

Other miscellaneous contributions include simulations, algorithms, theorems, and other applications not classified in the previous groups. These contributions can be useful for the research on V2X, I2X, and P2X communications, because they allow the optimization of parameters on models. An example of a simulation can be found in [112], whose model allows the simulation of intelligent transport systems and the introduction of elements such as traffic lights or roadside stations, among others. As the main advantage, this work provides results on the traffic flow of vehicles, communications, and RF emissions. Another example of outstanding work in algorithms is [113], whose authors present an approach that determines the optimal number of roadside units (RSU) required per area as a function of the vehicle density. Other works in theorems can also be found to provide network stability and scalability based on the topology of the communication flow [114]. In this sense, a novel model has been proposed to solve problems of shared resources by means of graphs, which offers better results than the traditional methods [115]. Finally, another notable work proposed the use of wireless energy in electric vehicles not only to transfer power, but also to support V2I communication in areas of high traffic density and data transfer rate [116].

### 3.3. Industry Interest in V2X, I2X, and P2X Communication

The consolidation of the industry concerning the R&D in communication technologies for vehicles, pedestrians, and infrastructure has been mainly motivated by the progress around the connected and/or autonomous vehicles (AVs). Some technological milestones in this regard have been the early functions of the e-Call system demonstrated in 1996, the remote diagnosis capability introduced in 2001, the network access device utilized for vehicle health reporting and turn-by-turn navigation in 2003, the only-data telematics used by Continental, and the 4G/LTE communication with WiFi hotspot access included by Audi, Volvo, and General Motors from 2014 [26].

An example of effort in the development of V2X communication is a car system that identifies traffic lights on public roads and communicates with a cloud server to predict how fast a vehicle should go to encounter green lights, as well as to predict waiting times when lights are red [117,118]. This technology, developed by Traffic Technology Service (TTS) for Audi, BMW, and Continental, allows more environmentally friendly driving and is less harmful for the vehicle components (e.g., tires). On the other hand, Honda has patented a system to communicate data (e.g., location, speed, etc.) from vehicles and pedestrians to alert and avoid accidents in path intersections [119]. Hitachi also worked on helping pedestrians to cross the road through a display-based system aboard vehicles that informs people and other nearby vehicles on future actions (e.g., give way and go) [120]. Moreover, Continental described a V2V/V2I communication system with redundant units to avoid shadow areas and enable the exchange of information [121]. In this line, Samsung developed an advanced information method and system to provide data from vehicles to all the surrounding actors (i.e., infrastructure, pedestrians, and cyclists) in critical areas of the road such as pedestrian crossings [122]. However, not all the communication systems patented are focused on avoiding accidents. This is the case of a vehicle used to record and manage road items in which a communication method based on queries and responses from/to a central node is described in [123].

Regarding the I2X communication, mostly techniques, methods, and communication systems in smart cities are implemented to make urban infrastructure safer and avoid accidents. For instance, an I2V communication system was used to detect pedestrians at zebra crossings and alert both nearby vehicles wirelessly and their drivers by acoustic and/or light signals [124]. In this sense, an apparatus was proposed to synchronize vehicles with pedestrians and facilitate crossings without accidents [125]. Also, a system that transmits auditory information for disabled people about road elements (e.g., traffic lights) when their personal devices are oriented to the infrastructure is described in [126].

Finally, there are several techniques, methods, and systems applied to P2X communication to interact between pedestrians or cyclists to everything. The state-of-the-art includes solutions such as a signaling device that alerts drivers about pedestrians or cyclists on the road through a luminous totem wirelessly activated by smartphones and personal devices [127]. In a similar way, a procedure to avoid accidents between pedestrians and vehicles based on the historical position—to determine the future location—and user context (e.g., age, response capacity, etc.) is used to alert about possible collisions by means of visual and/or acoustic signals [128].

## 4. Bibliographic Analysis

A more comprehensive and systematic bibliometric analysis has been conducted considering the online abstract and indexing service provided by Scopus^®^ from Elsevier. The reason for its choice—as opposed to others, such as the IEEE Xplore^®^ digital library, Google Scholar, ResearchGate, ArXiv, or DBLP—is that Scopus^®^ is considered the world’s largest scientific database. Furthermore, Scopus^®^ is available for free and has also been used in many previous bibliometric analyses [129,130,131,132].

The search range was focused on the period from 1997 to 2018 and performed with the following structure: TITLE-ABS-KEY (“*term_1*” OR “term_2” OR “*term_n*” AND “*term_i*” […] AND NOT “*term_j*” […]) AND (LIMIT-TO (SUBJAREA, “COMP”) OR LIMIT-TO (SUBJAREA, “ENGI”)) AND (EXCLUDE (PUBYEAR, 2019)). The publications were gathered to compare whole annual periods, limited to the categories of Computer Science and Engineering, then filtered to avoid the misuse of terms belonging to other disciplines (i.e., acronyms with various meaning) and finally processed using spreadsheets to sort the results. The publications in Scopus^®^ were evaluated considering the following factors: Number of manuscripts per year, source type, keywords, country, and affiliation of the authors. Note that the analysis made in this section has no potential bias, since all the manuscripts stored in Scopus^®^ have been entirely considered unlike the previous section. 

### Evolution of Mobile Devices, Sensors, and Intelligent Applications for Smart Cities

**Figure 4:**
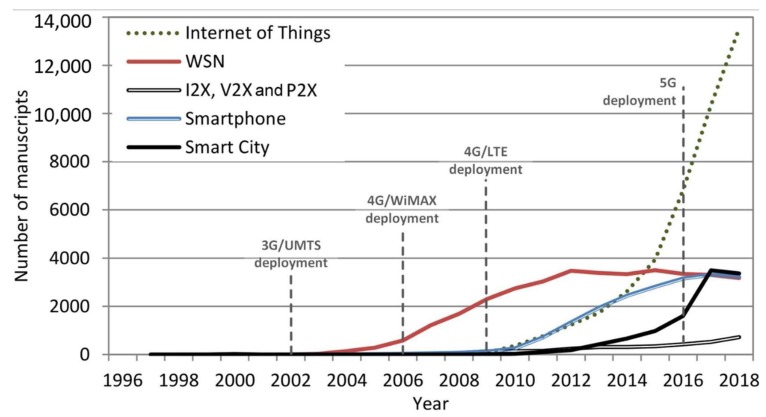
Evolution of key technologies for mobile devices, sensors, and intelligent applications in smart cities.

Regarding the technological context, the sample analyzed in this work included 10,867 articles within the “Smart City” category, 35,537 for “WSN”, 41,485 for “Internet of Things”, 19,475 for “Smartphone”, and 3422 for “I2X, V2X, P2X” and their variants (Figure 4). The survey showed a recent research in all fields in general with a major disruption of the “Internet of Things” (37.4%) over “WSN” (32.1%), “Smartphone” (17.6%), “Smart City” (9.81%), and “I2X, V2X, and P2X” (3.31%). A comparison between the evolution of the publications showed an outstanding correlation for “Smart City” versus “Internet of Things” (*r*^2^ = 0.967, *p* << 0.01). The analysis also showed a good correlation for “Smartphone” versus “I2X, V2X, and P2X” (*r*^2^ = 0.881, *p* << 0.05), “Internet of Things” versus “I2X, V2X, and P2X” (*r*^2^ = 0.878, *p* << 0.05), and “Smart City” versus “I2X, V2X, and P2X” (*r*^2^ = 0.809, *p* << 0.05). On the contrary, the bibliographic evolution showed a lower association in the publications of “WSN” versus “Internet of Things” (*r*^2^ = 0.279, *p* = 0.013) and “Smart City” versus “WSN” (*r*^2^ = 0.295, *p* = 0.008). This study suggests that the I2X, V2X, and P2X technologies are strongly related to smart cities, IoT, and smartphones. At the far side, WSN is less related with IoT and smart cities. 

**Figure 5:**
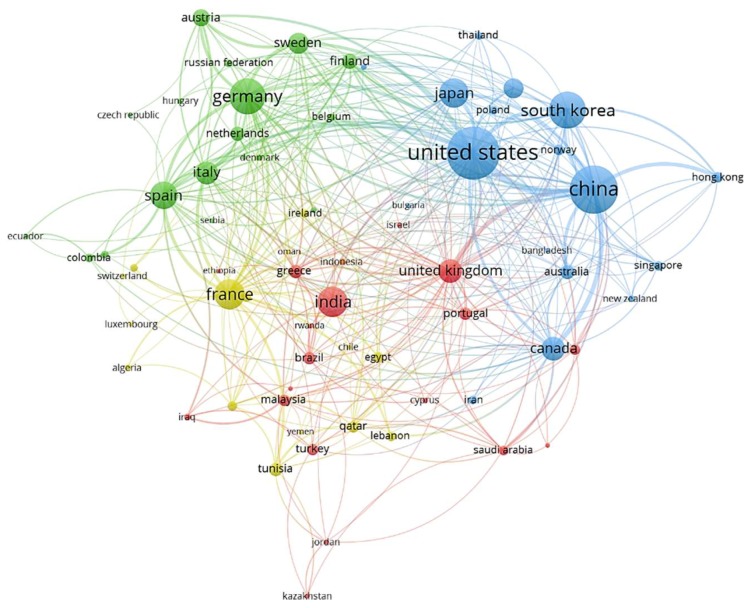
Cooperation between countries based on the publication co-authorship from 1996 to 2018.

An analysis on the publications per territory focused specifically on I2X, V2X, and P2X communications showed significant activity mainly in Asia (39.65%), Europe (32.25%), and North America (20.55%), followed by South America, Africa, and Oceania to a lesser extent (7.53%). A study on the most productive countries and their relationship was then conducted. To this end, VOSviewer was chosen as the software tool for creating, visualizing, and exploring maps based on network data [133]. The results are resumed in Figure 5 and Table 1 for which thesaurus files and the Lin-Log clustering technique were used to filter inconsistencies over a total of 80 countries. 

**Figure 6:**
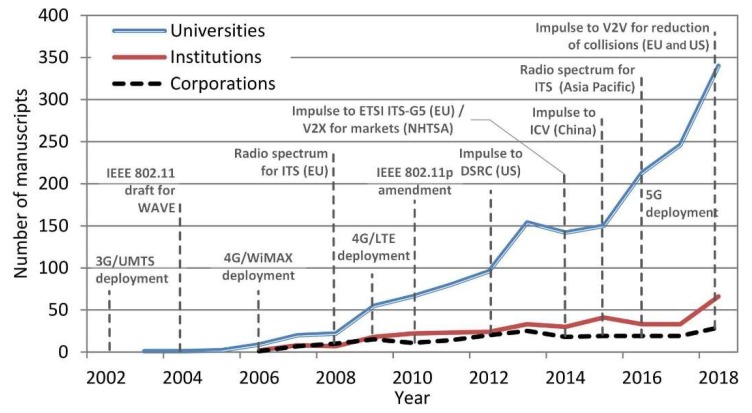
Bibliographic evolution of the contributions on I2X, V2X and P2X research per organization.

A study about the distribution of the organizations showed the leadership of universities (74.48%) over research institutions (15.85%) and private corporations (9.65%). The study on the correlation encountered a good relationship in the research carried out from 1996 to 2018, which confirms the close relationship between the different organizations (*r*^2^ = 0.869 and *p* << 0.05 for “Universities” versus “Institutions”, *r*^2^ = 0.717 and *p* << 0.05 for “Universities” versus “Corporations”, and *r*^2^ = 0.691 and *p* << 0.05 for “Institutions” versus “Corporations”). The study also encountered that the universities started the research in this field three years earlier than the other organizations (Figure 6). This, in addition to the greatest number of publications, suggests that universities mainly carry the weight of the investigation in this field. This can be derived from the most productive organizations resumed in Table 2 and Table 3, where the top of universities, institutions, and corporations is shown. 

**Figure 7:**
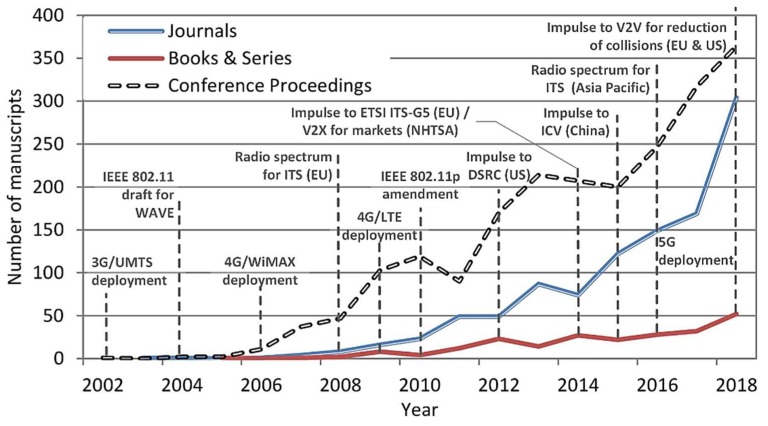
Bibliographic evolution of the contributions on I2X, V2X, and P2X research per source type.

An analysis on the source type of the publications (Figure 7) shows a major contribution through conferences (62.41%) followed by journals (30.96%) and books (6.62%). The study confirms a good correlation between the different sources, which suggests that a rapid and strong growth in conferences is also reflected in scientific journals and textbooks (*r*^2^ = 0.849 and *p* << 0.05 for “Conference Proceedings” versus “Journals”, *r*^2^ = 0.887 and *p* << 0.05 for “Conference Proceedings” versus “Book Series”, and *r*^2^ = 0.891 and *p* << 0.05 for “Book Series” versus “Journals”). The study on the document type found a total of 219 open access manuscripts (6.37%) as well as 41 surveys and short reviews (1.19%) over a total of 3439 manuscripts. These results reflect the weight of primary research versus secondary research.

**Figure 8:**
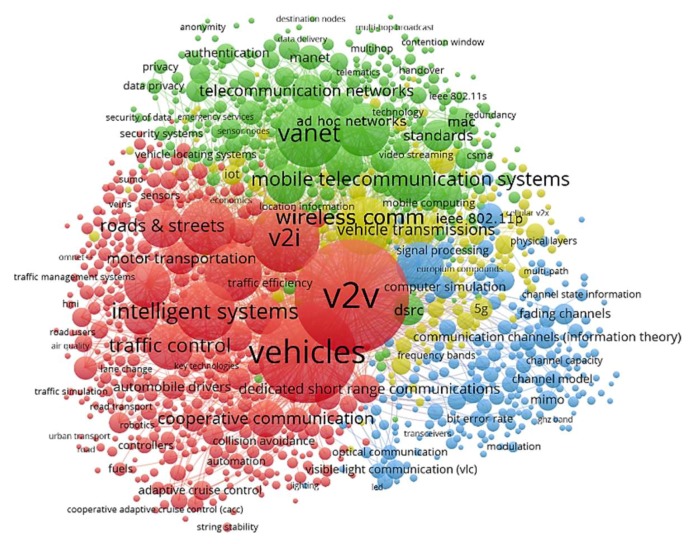
Co-occurrence of the keywords in publications on I2X, V2X, and P2X from 1996 to 2018.

An in-depth review on the co-occurrence of the keywords between the publications—both indexed by authors and publishers—is shown in Figure 8. The network visualization corresponds to the 1000 most popular terms over a total of 14,364 keywords within the 3422 manuscripts. Accordingly, the number of documents in which some representative keywords related to this survey appear resulted as follows: “V2V” (2476), “V2I” (896), “VANET” (872), “intelligent systems” (669), “V2X” (286), “IEEE 802.11p” (191), “LTE” (116), “MANET” (112), “WAVE” (103), “5G” (98), “VLC” (83), “IoT” (79), “sensors” (52), “D2D communication” (52), “WSN” (37), “I2V” (37), “IEEE 802.11s” (27), “smart city” (27), “mobile devices” (21), “smartphone” (21), “RFID” (21), “3G” (20), “WiMAX” (19), “Bluetooth” (15), “ZigBee” (15), “4G” (14), “LTE-V” (11), “V2R” (11), “IEEE 1609” (10), “V2P” (8), “RFID” (21) and “V2G” (7), among others. The frequency in which the terms appear allows us to assess the weight of research in this field.

## 5. Future Trends and Challenges

One of the most promising technologies and driving forces in the medium term is 5G. This access technology has been defined to work in three different usage scenarios: Mobile broadband (e.g., smartphones), machine-type communication (e.g., IoT sensors), and low-latency communication (e.g., industry 4.0 and connected vehicles). So, to boost the R&D on applications at the service of smart cities, 5G must provide higher reliability (up to 100%), data rates (20 Gbps), energy efficiency (10 mW/Mbps/sec), positioning accuracy (sub-meter range), quality of Service (QoS) for mobility (500 km/h), and lower latency (1 ms) than the previous cell-based technologies [134]. Although the 5G worldwide commercial launch is expected to be in mid-2020 after the approval of Release 16 by the International Telecommunication Union Radiocommunication Sector (ITU-R), there was in the past a strong early adoption in the form of pre-standards due to the great interest generated by the industry. This comprised service providers, hardware manufacturers, and national regulatory bodies (e.g., the 5G Technical Forum trial network of Verizon in 2010, the allocation of the 5G band by the FCC in 2016, or the 5G modems launched by Qualcomm and Intel in 2016-17). 

In this context, the connected, cooperative, and automated mobility (CCAM) has been identified as one of the main vertical services of 5G by 2020 [135]. With this purpose, some efforts in terms of cooperation to adopt and test the capability of the 5G networks for AVs are becoming visible as that from the 5G Automotive Association (5GAA) since 2016, the European Automotive and Telecom Alliance (EATA) since 2017, or some providers in South Korea (i.e., BMW, Ericson, and SK Telecom). This will provide the autonomous and/or connected vehicles with the possibility of implementing multi-tier convergence networks to address road safety and transport needs for a smarter future mobility, involving ITS, ubiquitous connectivity, AI systems, and transport as a service (TaaS) [10]. Nevertheless, the higher the cost of the 5G infrastructure to be deployed, the higher the losses of the 5G millimeter waves (i.e., indoor coverage up to 2 m), security, and privacy; these are some of the main issues yet to be solved [136].

Although 5G networks can certainly support a larger number of users at much higher data rates than 3G/4G networks, operating at millimeter wavelengths (i.e., 24–86 GHz) drastically reduces signal propagation in closed scenarios [137]. From a research point of view, this could be taken as an advantage rather than a handicap. That is, keeping interferences to a minimum allows multiple users and devices reuse the same spectrum at the same time. Therefore, future works seek to cover the need of users and devices to massively communicate wirelessly while having a high bandwidth. This opens up a new world of possibilities, particularly in the mobility context and for IoT-based HetNets connected indoors (e.g., smart fridges, intelligent thermostats, building health monitoring, or security systems). Hence, proposals beyond 5G must include radically new approaches to operate new high-speed data and energy-efficiency waveforms at frequencies above 90 GHz up to THz. This brings new opportunities to investigate around physical (PHY) layer technologies such as in antenna design, signal processing, information theory, and coding to optimize and reach speeds in the Tbps range [138].

Regarding the aforementioned handicap of 5G (i.e., it suffers severely from attenuation in indoor stages), 3GPP proposed in 2016 a new set of radio access standards to solve it by enabling wide-range cellular devices and services. This is the case of LTE-M and NB-IoT, two types of low power wide area networks (LPWAN) specially focused on machine-type communication approaches that need to transmit small amounts of data to the Internet at low cost—both in terms of hardware and service subscription—with a high battery life (e.g., traffic management, parking monitoring, or street lighting). The main features defined by 3GPP in Release 13 for LTE-M are 1 Mbps of maximum data rate, 10–15 ms of latency, and a cost of $10–15 per module, while for NB-IoT, these values are 250 kbps, 1.6–10 s and $5–10, respectively. Among the main drawbacks, no radio technology excels in all the features and the specific usage must be established on a case-by-case basis (i.e., there is no one-size-fits-all solution). So, while voice and roaming services are well supported in LTE-M, NB-IoT is more suitable for stationary devices. Furthermore, data-intensive and time-critical applications cannot be performed, for instance, in road safety, traffic control, or autonomous driving. In addition, the use of a licensed band—unlike other approaches such as the long-range WAN (LoRaWAN)—precludes coverage in regions without an Internet service provider (ISP) network infrastructure. As a result, although LTE-M and NB-IoT represent cutting-edge technologies aimed at creating new applications and business models, there is still much work to do in terms of adoption and maturity of this technology [139].

An example of development of more advanced services and business models at the expense of smart cities is the Internet of Things Application (IOTA), an accounting open-source technology that allows the secure and wireless exchange of information with special focus on the automotive sector [140]. In this area, the IOTA Foundation and the International Transportation Innovation Center (ICIT) have cooperated since 2018 to test AV solutions through its own IOTA node-based service network (i.e., Tangle). In addition, Volkswagen, Bosch, Orange, Accenture, and Schneider Electric have progressively joined this newfangled initiative, who plan to kick off an IOTA bill platform in Q1 2019, called DigitalCarPass, to automatically pay for certain services such as parking or recharging electric vehicles [141]. This young technology is considered as part of the future smart car economy model so-called mobility as a service (MaaS) also envisioned by BMW, GM, Ford, Hyperledger, and IBM to form one of the world’s largest consortia in crypto applications. As a result, IOTA has achieved a market capitalization of ~$800 million since 2017. Nonetheless, the lack of an intelligent contract system, the energy cost of the crypto mining process, the need for a decentralized network, and security are some of the main disadvantages that still need to be worked out at the moment [142].

An example on the need to solve issues related to security and privacy is the social IoT (SIoT), a paradigm of peer interaction through public networks where smart objects such as smartphones, vehicles, and RSUs socialize. SIoT has enormous potential to provide new high-level services using different access technologies (e.g., Wi-Fi, LTE, WiMAX, etc.), with vehicles being a leading exponent of application by exchanging information such as infotainment, traffic status, parking, routes, or weather [143]. Clear examples of Social Internet of Vehicles (SIoV) already operational in markets are a voice chatting system for dynamic vehicular communications (RoadSpeak), a car navigator that integrates traffic voice tweets (NaviTweet) or a mobility data sharing system based on social networks (Caravan Track) [4]. However, despite the enormous possibilities regarding social interaction between vehicles, infrastructure, and pedestrians, future works should focus on trusting social media and confronting ethical dilemmas in decision making. Therefore, social capabilities become the next barrier on the road to developing full self-driving cars to increase passenger safety, comfort, and efficiency, where intermediate steps from 2019 to 2027 will comprise awareness, sensing, cooperative, and synchronized driving [10].

As these skills become key for local decision-making (not only in vehicles but also in the interaction with infrastructure and pedestrians), new technological concepts and innovations must emerge. With this purpose, fog computing was introduced in 2014 by Cisco, and later promoted by the OpenFog Consortium, also joined by Dell, Intel, Microsoft, ARM, and Princeton University, as a technology to reduce the gap between cloud computing and IoT devices [16]. This new architecture allows things, apps, and devices to take advantage of the decentralization of the computing infrastructure and extend the services to the network border. Therefore, data, computation, and storage are distributed in the most logical and efficient place between data source and cloud. As a result, fog computing allows artificial intelligence (AI) to be brought to sensors. This paradigm, well-named as the Artificial Intelligence of Things (AIoT), will allow the performance of short-term analysis, which improves efficiency, reduces raw data traffic, and takes care of legal aspects about security and privacy (e.g., sensitive data subject to regulations in different countries). Popular fog computing applications include smart grid, smart city, smart buildings, software-defined networks, and vehicle networks. In particular, the benefits for vehicular fog computing (VFC) are large computation capacity, low latency, high mobility support, and low deployment cost. However, limitations in storage space, new computing architectures, and the operation of these systems in heterogeneous environments must still be investigated [144].

In relation to self-driving, future trends aim to protect the communications used in AVs against cyber-attacks to prevent the car occupants from suffering personal damage or data theft. To avoid potential security threats (e.g., such as in cybercrime-as-a-service), the automobile industry has set cybersecurity as a critical aspect to be included from the vehicle design to its decommissioning (i.e., to cover the full vehicle lifecycle) [145]. In order to achieve this goal, the car industry is currently involved in the development of the ISO/SAE 21434 standard to provide cybersecurity to road vehicles, which is expected to be published by 2020 [146]. Also with the goal of providing security to AVs, both Europe and the USA are promoting new strategies to include the auditing and certification of cybersecurity aspects beyond the own car functionalities. This includes validating the entire value chain from original equipment manufacturers (OEMs) and multi-tier suppliers to manufacturers [147]. Future steps should therefore be directed to consider the auditing and certification processes as an investment instead of a cost, also provided in the form of new industry approaches (e.g., automobile consortiums) to face the same security concerns [132].

## 6. Conclusions

Smart cities are scenarios of innovation, challenge, and opportunity to improve citizens’ lives, where intelligent transport and mobility are key pieces of interest. Autonomous and/or connected vehicles are an example of cooperative effort made in this area, with the communications to interconnect different road users such as vehicles, infrastructure, and pedestrians being the main topic of this paper. This literature review includes I2X and P2X, in addition to V2X, as the current R&D emerging technologies in this field. To this end, an analysis considering the 100 most representative documents of the state-of-the-art since the beginning of vehicular communications until today has been performed. To avoid disqualifying the most recent papers in the area, the followed methodology included 20.79% of the manuscripts published between 2000 and 2009, 45.54% published in 2010–2013 and 33.67% published between 2014 and 2018 (i.e., one third of the most significant papers were published in the last five years).

From the sample, we confirmed that governments, industries, and universities around the world closely cooperate to respond to needs in the development of regulatory laws, standards, and technologies. The key enabler aspects in the development of communications between vehicles, infrastructure, and pedestrians have been the spectrum regulations carried out around the world (e.g., the US Department of Transportation, the Federal Communications Commission in the USA, or the European Telecommunications Standards Institute in the EU), the IEEE 802.11p standard, the development of the 4G/LTE and 5G technologies, as well as the strategic development programs conducted in different countries (e.g., Made in China 2025). On the contrary, the main drawback for the progress of vehicular communications is the different step of adoption and the non-unification of the radio band locations in countries, which results into non-interoperable technologies across different territories.

Following the bibliographic analysis, we encountered that wireless signals and the improvement of road safety have been the main focus of the V2X, I2X, and P2X communications. In this sense, we found that most of the research and development in communications has centered on V2X (92.14%), I2X to a lesser extent (4.9%), and lastly on P2X (1.96%). These results suggest that the I2X and P2X communications have been barely studied, becoming an emerging field of research in recent years according to the publications. The study also analyzed the influence of one emerging technology over another from 1997 to 2018. Such is the case of the V2X, I2X, and P2X communication, which were strongly influenced by the progress of smartphones (*r*^2^ = 0.881), IoT technologies (*r*^2^ = 0.878), and smart cities (*r*^2^ = 0.809). As a result of the review, we found that the developments around the V2X, I2X, and P2X communications have been promoted mainly by universities (74.84%), followed by other research institutions (15.85%) and private corporations (9.65%). In this sense, the analysis encountered that most of the research was published in conferences (62.41%), followed by journals (30.96%) and books (6.62%).

On the other hand, as one of the conclusions of this review, it has been found that not all authors use the same expressions or acronyms to refer to the same concept. An example can be the indifferent use of car-to-car (C2C) versus vehicle-to-vehicle (V2V) communication, or car-to-everything (C2X) versus vehicle-to-everything (V2X) communication. In order to help solve these misuses, this work proposed a taxonomy to homogenize terms, avoid errors and provide long-term stability for the V2X, I2X, and P2X communication and their variants. In addition, this classification aims to contribute a useful framework that helps save time and effort to researchers and developers when designing future specifications and technical requirements around these communications.

As for future research, we confirmed that one of the most promising and leading technologies in the medium term is 5G. Although 5G networks will provide better performance than 3G/4G networks in general (i.e., reliability, data rate, power consumption, positioning, QoS, and latency), the cost of the 5G infrastructure, the signal loss of the millimeter band, and some aspects on security and privacy are some of the main concerns yet to be solved. In the meantime, other noteworthy proposals beyond 5G, such as LTE-M, NB-IoT or new ways of waveform at frequencies up to THz, have to prove their worth. This would open up a new world of possibilities to more advanced services and business models. In this sense, the Internet of Things Application (IOTA) or the Social Internet of Vehicles (SIoV) are current examples from the automotive sector aimed at increasing the safety, comfort, and efficiency of passengers in the path to developing fully self-driving cars by 2027.

## Figures and Tables

**Table 1:** Most productive countries in I2X, V2X, and P2X research from 1996 to 2018.

R	Country	MP	MP (%)	CA	IC	CR	CR (%)
1	USA	667	19.24	344	46	7856	31.55
2	China	538	15.52	340	30	3573	14.35
3	S. Korea	332	9.57	101	22	1266	5.08
4	Germany	302	8.71	175	32	3600	14.46
5	France	221	6.37	167	36	1690	6.78
6	India	219	6.31	26	14	676	2.71
7	Japan	207	5.97	72	19	919	3.69
8	Spain	178	5.13	133	29	2337	9.38
9	Canada	139	4.01	109	29	1465	5.88
10	UK	133	3.83	131	39	1706	6.85

R: Ranking position; MP: Manuscripts published; CA: Co-authorships with other researchers; IC: International collaborations; CR: Citations received.

**Table 2:** Most productive institutions in I2X, V2X, and P2X research from 1996 to 2018.

R	Organization	CL	MP	MP (%)	NC	CR	CR (%)
1	Beijing Univ. Posts and Telecom. (BUPT)	U	66	1.92	47	345	1.38
2	Beijing Jiaotong Univ. (BJUT)	U	60	1.74	50	455	1.83
5	Univ. Politècnica de València (UPV)	U	39	1.13	27	350	1.41
6	Hanyang Univ.	U	39	1.13	12	95	0.38
7	Electronics and Telecom. Research Institute (ETRI)	I	38	1.10	21	156	0.63
8	Tsinghua Univ.	U	38	1.10	43	303	1.22
15	DeutschesZentrum fur Luft-Und Raumfahrt (DLR)	I	31	0.90	29	131	0.53
25	ConsiglioNazionaledelleRicerche (CNR)	I	24	0.69	18	200	0.80
27	Qatar Mobility Innov. Center (QMIC)	I	24	0.69	15	233	0.94
30	Instituto de Telecomunicações (IT)	I	22	0.64	29	428	1.72

R: Ranking position; CL: Classification; U: University; I: Institution; MP: Manuscripts published; NC: Collaborations with other organizations; CR: Citations received.

**Table 3:** Most productive enterprises in I2X, V2X, and P2X research from 1996 to 2018.

R	Organization	MP	MP (%)	NC	CR	CR (%)
3	General Motors (GM)	49	1.43	56	641	2.57
22	Volvo	26	0.76	54	322	1.29
40	Toyota Info Tech. Center	19	0.55	21	235	0.94
45	Volkswagen AG	18	0.52	23	137	0.55
57	Forschungszentrum Telekom. Wien (FTW)	16	0.47	16	500	2.01
60	DENSO Corporation	16	0.47	8	9	0.04
61	NXP Semiconductors	16	0.47	4	30	0.12
64	Renault	15	0.44	20	46	0.18
81	Ford Motor Company	13	0.38	33	49	0.20
93	Huawei Technologies Co., Ltd.	12	0.35	18	89	0.36

R: Ranking position; MP: Manuscripts published; NC: Collaborations with other organizations; CR: Citations received.

## Abbreviations

3G	Third Generation
4G	Fourth Generation
5G	Fifth Generation
5GAA	5G Automotive Association
AV	Autonomous Vehicle
C2C	Car to Car
C2I	Car to Infrastructure
C2X	Car to All/Everything
CSMA	Carrier Sense Multiple Access
C-V2X	Cellular V2X
D2D	Device to Device
EATA	European Automotive and Telecom Alliance
eV2X	Enhanced V2X
EWM	Emergency Warning Messages
FCC	Federal Communications Commission
FOV	Field of View
HetVNET	Heterogeneous Vehicular Network
I2I	Infrastructure-to-infrastructure
I2P	Infrastructure-to-pedestrian
I2V	Infrastructure-to-vehicle
I2X	Infrastructure-to-everything
ICT	Information and Communication Technologies
ICT	Intelligent Connected Transport
ICV	Intelligent Connected Vehicle
IEEE	Institute of Electrical and Electronics Engineers
IN-V	In-vehicle
ITS	Intelligent Transport Systems
IoT	Internet of Things
IOTA	Internet of Things Application
ITU	International Telecommunication Union
ITU-R	ITU Radiocommunication Sector
LoRaWAN	Long Range WAN
LOS	Line-of-sight
LPWAN	Low Power Wide Area Network
LTE	Long Term Evolution
LTE-M	LTE Machine
LTE-V	LTE for vehicles
M2M	Machine-to-machine
MANET	Mobile Ad Hoc Network
MBMS	Multimedia Broadcast Multicast Service
NB-IoT	Narrow Band IoT
NDN	Name Data Networking
P2I	Pedestrian-to-infrastructure
P2P	Pedestrian-to-pedestrian
P2V	Pedestrian-to-vehicle
P2X	Pedestrian-to-everything
QoS	Quality of Service
R&D	Research and Development
RFID	Radio Frequency Identification
RSU	Roadside Unit
SDN	Software-defined Networking
TaaS	Transportation as a Service
TDMA	Time Division Multiple Access
V2B	Vehicle-to-broadband Cloud
V2C	Vehicle-to-cloud
V2D	Vehicle-to-device
V2G	Vehicle-to-grid
V2H	Vehicle-to-home
V2I	Vehicle-to-infrastructure
V2N	Vehicle-to-network
V2M	Vehicle-to-motorcycle
V2P	Vehicle-to-pedestrian
V2R	Vehicle-to-road
V2S	Vehicle-to-sensor
V2V	Vehicle-to-vehicle
V2X	Vehicle-to-everything
VANET	Vehicular Ad Hoc Network
VFC	Vehicular Fog Computing
VLC	Visible Light Communication
VSN	Vehicular Sensor Network
WAVE	Wireless Access in Vehicular Environments
Wi-Fi	Wireless Fidelity
WSN	Wireless Sensor Network
WiMAX	Worldwide Interoperability for Microwave Access

## Appendix A

**Table A1:** Technological profile of the 100 most cited publications in the state-of-the-art.

Reference	Type	Case of Use	Players	Technology	Application	Country	Year
[12,13,28,37,43,55,56,59,63,66,71,72,73,74,75,86,93,94,95,98,104,107,108,113,115,121]	V2V and V2I	Survey, Analytical model, Road safety, Security, Others, Patent	Vehicles and Infrastructures	2G, 3G, 4G, LTE, Cell band, 802.11p, 802.11 standards, WiFi, IEEE 1609.4, VLC, WiMax, 802.11b/g and WLAN-based	VANET, ITS, Communications and Smart cities	UK, India, US, Germany, Singapore, China, Australia, France, Canada, S. Korea, Spain, Japan	2008, 2009, 2010, 2011, 2012, 2013, 2014, 2015, 2016, 2017
[29,30,31,50]	V2I and V2R	Survey, Analytical model	Vehicles, Infrastructures and Road	802.11, 802.11p, 802.11e-based and 802.11a-based	Communications, VANET and ITS	US, Germany, US, France	2003, 2007, 2014
[32,33,34,35,36,39,40,41,42,44,45,46,47,49,51,52,55,57,58,60,61,62,70,76,77,78,79,80,81,82,84,88,92,100,105,106,109,110,111,114,120]	V2V	Analytical model, Survey, Road safety, Security, Multimedia, Patent	Vehicles, RSUs and APs	802.16, 802.11 standards, 802.11p, IEEE 1609.4, Visible light, 2G, 3G, 4G, LTE, WiMax, RFID and Bluetooth	Communications, ITS, VANET and Smart cities	US, UK, Sweden, China, Belgium, Germany, Italy, Austria, S. Africa, Canada, Thailand, Spain, Portugal	2004, 2005, 2007, 2008, 2009, 2010, 2011, 2012, 2013, 2014, 2015, 2016, 2017
[38,48,68,83,87,89,96,102,103,116,123]	V2I	Analytical model, Security, Road safety, Patent	Vehicles, Infrastructures and RSUs	802.11 standards, 802.11p, SFN, 3G, RFID	Communications, ITS, VANET and Smart cities	Spain, UK, Canada, Taiwan, Germany, US	2008, 2009, 2010, 2012, 2015, 2011
[53]	C2C	Analytical model	Vehicles	5 GHz, 24 GHz and 76 GHz	ITS and Communications	Germany	2011
[64,65,69,101,112,117,118,119,122]	V2X	Analytical model, Survey, Security, Simulation, Patent	Vehicles, Pedestrians, Infrastructures, etc.	802.11p, 3GPP, 4G, LTE, Cell band, WiMaxand 802.11 standards	Communications, ITS, VANET and Smart cities	China, S. Korea, Canada, Netherlands, US	2011, 2015, 2016, 2017
[67]	V2V, V2I and V2C	Survey	Vehicles, Infrastructures and Cloud	802.11 standards and LTE	Communications, ITS, VANET and Smart cities	China	2015
[99]	V2V, V2I and I2V	Security	Vehicles and Infrastructures	-	ITS, VANET, Communications and Smart cities	Netherlands	2015
[85,124]	I2V	Road safety, Patent	Infrastructures and Vehicles	AM or FM radio, GPS, RFID and Cell band	Smart cities and ITS	Spain, US	2010
[90]	V2C and V2V	Road safety	Vehicles and Cloud	-	ITS, VANET and Smart cities	China	2016
[91]	M2M	Road safety	Smartphones and Cloud	802.11 standards	Smart cities, Smartphones and IOT	India	2012
[97]	C2P	Survey	Vehicles and Pedestrian	802.15.4, 802.11p, 3G and 4G	ITS and Smart cities	China	2017
[119]	V2P	Patent	Vehicles and Pedestrians	802.11 standards	Smart cities and ITS	US	2016
[125]	I2X	Patent	Vehicles, Infrastructures, Pedestrians, etc.	LAN, MAN, WAN, Cell band, WLAN, Bluetooth andWiMax	Smart cities and ITS	France	2015
[126]	I2P and I2V	Patent	Infrastructure, Pedestrian and Vehicles	High frequency carrier	Smart cities	France	2004
[127]	P2I	Patent	Pedestrian and Infrastructures	Wireless, GPS and Bluetooth	Smart cities and ITS	Spain	2016
[128]	P2V	Patent	Pedestrian and Vehicles	Cell band, WLAN, PAN andWiMax	Smart cities and ITS	Germany	2010
